# Abstracts and Gleanings

**Published:** 1886-02-20

**Authors:** 


### ABSTRACTS AND GLEANINGS.

**Case of Dystocia.**—(Maryland Medical Journal.)

CASE I.—Mrs. B. W., American, white, æt. 25 years, 1 para. Labor at full term December 16th, 1885. Membranes ruptured 10 p. m., although real pains were not experienced until midnight. Os dilated regularly and completely by 4 a. m. December 17, Diag. L. S. I. A. breech movable above the superior strait. Descent failing, chloroform, manual extraction, Elliott's forceps, were resorted to by the attending accoucher, Dr. S., but with an unsuccessful result. Professor Miltenberger being summoned, arrived 7.45 a. m., and with the patient under chloroform tried (a) manual extraction, (b) to pull down a foot, likewise unsuccessfully.

I was added to the consultation 8.30 a.m., and found the patient partly under chloroform, the os uteri dilated and retracted up over the presenting breech, which was still movable at the pelvic brim, and moreover the brim seemed to be slightly contracted in its antero-posterior diameter.

For fully half an hour did I make fruitless attempts at manual extraction. It was difficult to hook the finger tips in the groin and the invariable result of traction thereon was only to bring one hip a little down while the other ascended correspondingly above the superior strait. I then tried my utmost to reach a foot, but as both legs were thrown up straight along the anterior surface of the child, with the feet quite in the fundus ureri, and the womb clasping the child very tightly, this attempt also proved a failure. The cord could be felt pulsating fairly well, although the heart sounds could not be heard by external ausculation. Various methods to pass the fillet failed. Remembering what favor Lusk (edition 1885) regards the use of Tarnier's forceps in difficult breech labors, when the breech is below the pelvic brim, and having myself already fruitlessly tried all safe means to effect delivery, I thought that even here with the breech above the brim, the forceps (almost as a last resort prior to employing the dangerous blunt hook,) if cautiously used, might be permissible.

The instrument employed was the Lusk-Howard modification of Tarnier's forceps, which was easily introduced and locked, and at first seemed to hold well, but finally with stronger tractions slipped without the slightest damage. I again resumed my former efforts at manual extraction with the finger hooked in the groin, but with the same unsuccessful result.

A second time did I attempt to reach the feet, and finally with much difficulty and at the risk of rupturing the uterus, I succeeded in bringing down one foot without doing any damage. As modate traction upon this did not draw the breech through the brim, I brought down the other foot, after which extraction of the trunk was easily effected. Delivery of the arms, which were thrown up alongside of the head, and of the after-coming head, was a task of some little difficulty, and at last the patient was delivered, with the perineum uninjured, of a large 10 lbs., well-formed, asphyxiated female child, presenting a few bruises about the groins, after at least one hour's .continuous manipulations by myself, not including the previous work of the other two physicians. The child was born limp and with pulseless cord, but as the heart could be felt to flutter feebly beneath the heart the thoracic wall, and as a slight gasp was observed, I persevered in my attempts to induce respiration and finally succeeded by the aid of Schultze's method.

The third stage of labor was soon terminated, the perineum was uninjured, some trouble arose on the eighth puerperal day from which the patient is now out of all danger. * * * *

The following remarks may serve to indicate the line of discussion for the society, which, as best illustrated by the present case, I would suggest might aptly fall under two principal subjects, viz.:

1. Barnes' method of decomposition.
2. The obstetric forceps, in difficult breech labor.

I purposely confine myself to a few citations from the best authorities, upon these two practically important points, not only as illustrated by the case, but also for the additional reason, which I trust you will bear in mind, that this report is not intended to be sufficiently comprehensive to embrace a discussion of all the causes and management of every possible difficulty that may be met with in presentations of the pelvic extremity.

Presentations of the breech alone, unaccompanied by the inferior fœtal extremities, *frank* breech (Pinard), *mode des fesses* (Charpentier, &c.) have been particularly studied by the French; Tarnier, Chantreuil, Charpentier, Budin, Pinard, Cantacuzene, Lefour, Olivier, etc, "and in the writings of these different authorities are raised many points of interest."

"Both Lefour and Olivier agree, that the presentation of the pelvic extremity where the breech or nates alone offers, is that which most frequently requires the intervention of the accoucheur, and they both acknowledge the danger of that intervention for the child, whether it be either manual or instrumental."*

Most authorities attribute the cause of the dystocia in such cases to the fact of the breech being an inefficient dilator as compared to the vertex, its imperfect adaptability to the inferior uterine segment, hence the slowness of the first stage, the early rupture of the membranes before complete dilatation of the os, and escape of the liquor amnii with possibly prolapse and subsequent compression of the funis, either between the child's body or after coming head and the pelvic wall, and also extension of trunk, arms, head, occurring spontaneously or produced by art.

Futhermore, Tarnier† says: "The difficulty consists in the production of lateral flexion of the body by which disengagement is usually effected, and this is because the inferior extremities thrown up along the whole length of the trunk, form *splints* to the fœtal vertebral column, and maintain it rigid." ‡

---

*Charpentier: "Taite Practique des Accouchements:" Edition 1883, vol. II pp. 856-7.

†Tarnier and Chantreuil: ' Trate de Arts des Accouchements. Edition 1882, vol. I, pp. 669-70.

‡Charpentier, vol. I, p. 448, says: "That both engagement and disengagement and of the breech either do not occur at all, or occur too slowly."

As regards the management of such cases, the difficulty of prehension either manually or instrumentally is admitted by all, hence, I bring the above teaching of the most recent French authority before the notice of the society as a very practical point that may indicate the proper line of treatment. For, if this teaching be correct, I think it would naturally lead us to adopt the plan so strongly advocated by Barnes in the last edition of his able work on obstetrics, published in 1885.

Barnes advises in breech presentations either at or above or below the superior pelvic strait, whether the legs are (*a.*) thrown up alongside the trunk as in the present case ; or are (*b.*) flexed down by the breech ; whenever interference becomes necessary for the delivery of the breech, the proper thing to do is to bring down a foot or feet and thus decompose the wedge, after which extraction is facilitated. "We have on several occasions brought a live child into the world by this proceeding when forceps, hooks, and various other means had been tried in vain ; this, we repeat it emphatically, is the right thing to do in the first instance. * * * It is of no use to attempt to bend the leg by acting upon the thigh or knee, the finger must be applied to the instep and therefore carried nearly to the fundus of the uterus ; no ordinary case of turning involves passing the arm so far." ‖

Nevertheless, if the finger can be hooked in the groin, no matter whether the presenting part be at or below the superior strait, for my own part, I would not utterly ignore the practice of making careful, yet strong traction upon the breech, aided by external pressure over the opposite extremity of the fœtal ovoid ; and, hence, I think this method is at least deserving of a fair trial in appropriate cases, especially where, as in the present, Barnes' method could not at first be performed.

In speaking of the management of this variety of breech presentation, Leishman§ says: "It is sometimes possible, and the more so when the breech is high in the pelvis, to break up the presentation by pulling down one leg." Playfair¶ says: "If, however, the legs be extended on the abdomen, it will be necessary to introduce the hand and arm very deeply, even up to the fundus of the uterus, a procedure which is always difficult and which may be very hazardous ; nor do I think, that the attempt to bring down the feet can be safe when the breech is low down and fixed in the pelvic cavity." I am strongly inclined to agree with this last statement, although I have yet to test it clinically.

Zweifel* advises : when the breech is movable above the brim to pull down the anterio foot; and adds, that when the breech has entered the true pelvis this plan should be rejected because of the danger of fracturing the thigh ; here we must make extraction directly upon the breech, (*a.*) manually, if possible ; (*b.*) instrumentally, using first the fillet, second the blunt hook.

---

‖ "Barnes: System of Obstetric Medicine and Surgery." Edition 1885, pp. 809, et seq.
§ Leishman : Edition 1879, pp. 326.
¶ Playfair. Edition 1885, pp. 319.
* Zweifel: Lehrb d. Operative Guburtshulfe; Edition 1881, pp. 216.

Schroeder† says: "We should not give up all hope of bringing down a foot even when the breech has entered the pelvis, for here, aided by position of the woman, while one hand manipulates internally, we can sometimes succeed in pushing up the breech and then decompose the wedge; and I am inclined to think this should be tried before resorting to instruments.

Lusk‡ says: "In a few cases, influenced by Barnes' teachings, I have under the most difficult conditions succeeded in bringing down a foot; nevertheless, I would advise the utmost caution in practicing the method, and that in all cases the operator desist the moment address fails and force becomes necessary."

**The Treatment of Pleurisy in the Bellevue Hospital.**—(London Medical Times) Dr. S. Mitchell, writing in the Therapeutic Gazette for November, states that about 150 cases of pleurisy are treated annually. It is rare to meet with true cases of acute pleurisy, except when it occurs in patients while in the hospital. When a case, however, is seen within the first few hours, opium is given, usually as Dover's powder or as Majendie's solution hypodermically, which, besides relieving the pain and nervous manifestations, to some extent checks the determination of blood to the pleura. The bowels are opened by salines, and mustard or turpentine applied to the chest. The pain caused by the movement of the chest is greatly relieved by strips of adhesive plaster. Tincture of aconite is given in half-minim doses every fifteen minutes for two hours, and afterwards every two hours until the pulse shows signs of becoming feeble. Quinine in doses of 10 grains every six hours is given during the first twenty-four hours. When the state of effusion occurs the patient is made to take freely of bitartrate of potash solution as a diuretic, the saline cathartics are continued, and iodine is applied locally. Another form of local application, which is a favorite with some, is the punctuated cauterization with Paquelin's cautery every other day. Tonics are given and continued into the third stage, the following formula being that usually prescribed: Strychniæ sulph., gr. i; liq. pot. arsenit, ʒ ij; cit. ferri et quiniæ, ʒ iv; glycerinæ aq. cinnam part. equal, ad ℥ viii; a dram after meals. With this is often given an ounce of whisky three times a day. A dram of the following mixture is also given occasionally to allay the cough: morph. sulph., pot. cyanidæ, gr. ij; syr. tolut., syr. pruni. vir. part. equal, ad ℥ ij. Blisters are seldom employed. When the effusion is great enough to cause much dyspnæ parancenticis is performed at the mid-axillary line of the sixth interspace, the fluid being withdrawn slowly and arrested at the moment when the patient begins to cough or feel other unpleasant symptoms. In the chronic form of the disease the patient is put on diuretics, tonics, and mild cathartics, and counter-irritation is kept up by Corson's paint, made of ol. tiglii. ʒ ij; ætheris, ʒ iv; tr. iodin. co., ad ℥ ij. This painted on every morning produces a crop of pin-head blisters.

† Schroeder: Lehrb. d. Geburtshulfe: Edition 1880, pp. 309.
‡ Lusk: Edition 1885. pp. 378.

with very little annoyance. When absorption does not occur, this has seemed in many cases to become stimulated by aspiration, a few drams of liquid being removed by means of a hypodermic syringe, this often rendering paracentesis unnecessary.

**Supra-Pubic Aspiration for Retention of Urine.**—(New York Medical Record) Dr. W. McChesney, of Wauconda, Illinois, reports the case of a man, aged 67, who suffered from stricture, the result of gonorrhœa contracted some twenty years ago. On attempting to pass water on the evening of June 5th, he was unable to do so, and getting no relief in the morning, sent for the writer. Repeated efforts were made to pass Nos. 6, 8 and 10 gum catheter, but without success; examination per rectum revealed the presence of a greatly enlarged prostate. Before proceeding to puncture, which operation was considered inevitable, the writer called in consultation Dr. Galloway, of Libertyville, who suggested the administration of chloroform and another attempt at catheterization before puncturing. This was tried without success and the bladder was then punctured above the pubes with a small trocar, about three pints of dark-colored urine being removed.

The canula was retained in position by means of threads of adhesive-plaster. "I considered this preferable to repeated tapping with the trocar, as I could see no prospect, immediate at least for the relief of the retention through the urethra. During the next two weeks the bladder was washed out twice daily with a solution of Squibb's boric acid, two drachms to the pint. This sufficed to keep the organ in a healthy condition, as far as could be judged by the appearance of the urine. I attempted to pass a catheter every other day, but found it impossible to do so. In the meantime the opening into the bladder occupied by the canula had enlarged sufficiently to allow of the introduction of a small drainage-tube, which greatly facilitated the process of irrigation.

"Acting upon the advice of Muflass, of Wheeling, who saw the patient with me, I now gave large doses of potassium iodide, but as the drug was not tolerated by the stomach it was soon discontinued. After repeated trials with the catheter, with every variety of curve, I discharged my patient. I saw him last week and he is as well as usual. Irrigation with the boric acid solution every other day keeps the urine clear."

**A New Method for the Removal Foreign Bodies from the Nose.**—(New York Medical Record) Dr. D. Bryson Delavan, of New York, sends us the following: "The presence of a foreign body in the nasal cavity is usually attended with marked swelling of the mucous membrane. Its extraction by any of the means in common use is accompanied with pain, often of great severity, and is often followed by copious hemorrhage. The swelling offers, of course, a serious obstacle to the extrusion of a hard body, while one which has increased in size from the imbibition of water becomes all the more firmly impacted. Hence, in attempting the removal of the body, more or less laceration of the membrane is likely to occur. The pain, with difficulty tolerated by an adult, causes a child to become in almost every instance unmanageable, so that an anæsthetic is required. The hemorrhage is usually controllable after the lapse of a few minutes, but may, meanwhile, cause considerable annoyance. From our knowledge of the physiological action of cocaine upon the nasal mucous membrane, it is evident that, by its use in these cases, all of the above difficulties may be overcome; for applied to the nose, the mucous membrane becomes strongly retracted, the sensibility to pain lost, and the blood-vessels exsanguinated. Thus, the calibre of the fossa is greatly widened, the irritation and consequent resistance done away with, hemorrhage prevented, and the removal of the foreign body thereby greatly facilitated. To carry out the method, the occluded nostril should first be cleansed with a spray or a gentle current of some lukewarm alkaline solution, after which a four per cent. solution of cocaine should be applied to the mucous membrane. When its effect has become complete, the extrusion of the body should be attempted by directing the patient to blow forcibly through the affected nostril. Failing in this, it should be drawn out by some suitable instrument. Should the patient be too restless to make this practicable, an anæsthetic may still be administered. In case of invasion of the frontal sinus or antrum of Highmore by insects or larvæ, cocaine should be applied to the membrane before the administration of chloroform or ether, in order that the canals leading to these cavities may become as patent as possible, and thus the vapor of the anæsthetic be admitted very thoroughly to the intruder's presence. The insensitiveness of the membrane produced by the cocaine will, in these cases, certainly add to the comfort of the sufferer should it be necessary to inject, or still better, to spray the nose with chloroform."

**Diphtheria Cured by Tolu Varnish.**—Richard Lord, M. D., in British Medical Journal: M. O. L——, aged thirteen, complained at 2 o'clock on November 10 of malaise. She was in bad spirits, owing to the death of one of her schoolfellows from diphtheria. A saline aperient was ordered, and taken in the evening. Next morning at 7 o'clock she said she felt "all right," but complained of sore throat.

Upon examination, thick, well-formed, grayish-looking patches, rather smaller than a florin, but of oval shape, with gangrenous edges, were seen over the right tonsil, and on the right posterior pillar of the fauces. At 5 o'clock in the afternoon the patches had somewhat increased, and two small patches were seen on the other side. The diphtheritic spots were covered with tolu varnish, as recommended in Dr. Morell Mackenzie's work. Tincture of perchloride of iron, with glycerine and chlorate of potash was prescribed as a constitutional remedy. The patient expressed herself greatly relieved by the varnish, and I applied it twice a day, instead of once, as advised by Dr. Mackenzie. In about forty-eight hours from the time when it was first seen, the membrane began to disappear, and on the evening of the fourth day not a trace of it remained.

I may add that it is important that the fauces and tonsils should be fiist well dried with blotting-paper. The solution can be most conveniently applied with a camel's hair-pencil fixed into a long wooden penholder, as supplied by Messrs. Maw. The method of treatment which I have found so successful in this case, being, I believe, little known, I think I shall be doing a service to my brother practitioners in placing it on record.

**Fatal Dose of Sulphate of Quinine.**—M. Baills, a French army surgeon, (Chemist and Druggist) reports an extraordinary case of quinine-poisoning, which is quoted with due comment in the Union Pharm.

A corporal, returning from drill, visited the infirmary and asked a Zouave in charge to give him a purgative. The latter agreed, and decided to take a dose himself as well. Intending to take the medicine from a bottle oontaining a solution of sulphate of sodium, he took in mistake a bottle containing a solution of sulphate of quinine, and both soldiers drank off a tin mugful of the liquid. It is calculated that the dose taken by each was about 180 grains!

Both soldiers went about their ordinary occupations until about half an hour after taking the medicine. Then commenced buzzings in the ears in one case, and stomach cramps and vomiting in the other. Both patients were placed in one room and attended by M. Baills, who reports the symptoms.

There were great pallor, dilated pupils, eyes projecting and fixed, short and anxious respiration; diminution of the respiratory report on auscultation. Reduction of temperature appreciable to the hand; pulse small, irregular, and at times scarcely appreciable. Beating of the heart similar. In fact, the circulatory system generally seemed particularly affected. Some burning pain in the throat, and much thirst. No nervous agitation nor convulsive movements; much buzzing in the ears; intelligence perfectly preserved. One of the patients declared that he could hear nothing, and that his ears seemed likely to burst.

Strong emetics were administered, and free vomiting was excited. Strong infusions of coffee were administered, both as a beverage and as an enema. Cold water was applied to the head, and mustard to the chest. The Zouave was very anxious about his comrade. In a second fainting fit, which occurred about two hours after taking the quinine, the Zouave died suddenly. The corporal was in the hospital a week, and completely recovered.

**Alkaloid Pomegranates.**—(American Druggist, Dec., 1885.) A crystalline body has been obtained in minute quantities from the rind of pomegranates, which, when placed upon the tongue or other portions of the mucous membrane, appears to cause a paralysis of local sensation after the manner of cocaine.

**Cocaine in Gonorrhœa.**—(Western Druggist) Bono (Gaz. delle Cliniche I, 1885) states that injection of a few drops of a two per cent. solution of cocaine rapidly relieves the chordee and pain in urinating following upon gonorrhœa.

**Cessation of Menstruation.**—W. E. Hughes, M. D., of Portage, Wood County, Ohio, (in the Medical Age), gives the following general rule for ascertaining the menopause in a particular case: Find out at what age menstruation began in any particular case; subtract this from seventy, and divide by two. The result is the number of child-bearing years. Add this to the age at which menstruation began, and it will give the age at which it should cease. For instance:

| | |
|---|---|
| Years of life | 70 |
| Menstruation began at | 16 |
| Half of which is | 27 |
| (The child-bearing period.) | |
| Add the commencing age | 16 |
| Giving age at which it ceases | 43 |

Bearing in mind the facts that those who begin early, cease early, and those who begin late, cease late, we should have the following table:

Beginning at 12, ceases at 41.
Beginning at 14, ceases at 42.
Beginning at 16, ceases at 43.
Beginning at 18, ceases at 44.
Beginning at 20, ceases at 48.

Although this table has been found in the main correct, allowance must be made for causes producing variations. It is not sent forth as infallible; but it will be found to "hit" nine times out of ten. It is doubtful if any trace of the idea will be found in works bearing on the subject, and it is believed to be original with the writer.

**A Parturiometer.**—(Weekly Medical Review) Dr. Henry Leaman exhibited a parturiometer before the Philadelphia Medical Society. He stated that this instrument can be placed inside of the cervix, in contact with the membranes; or, if the membranes are broken, against the advancing part of the fetus before full dilatation of the cervix. It can be used at the superior strait with ease. In the various conditions in which it has been employed, its registration has corresponded with experience, and rendered observation more accurate.

The parturiometer indicates when it is proper to break the membranes; when the cervix is fully dilated; when the application of instrument becomes necessary. By observing, in cases when the resistance is nil or at a minimum, and then again, when it is at a maximum, the amount of force lost may thus be arrived at. Also, by close observation, we may finally arrive at the separation of the true uterine force as distinct from anything else, and thus a more accurate knowledge of the physiological action of the uterus may be obtained.

This instrument promises to render the attendance of labor cases more exact and scientific.

**Forcible Injections in Intestinal Obstruction.**—(Journal American Medical Association) In the American Journal of the Medical Sciences, for January, 1886, Dr. H. Illoway, of Cincinnati, reports four cases of intestinal obstruction treated by the forcible injection of water, with three recoveries and one death. The injections were made with a powerful force-pump, and repeated two, three or more times, as necessary. The post-mortem examination in the fatal case showed that a knuckle of the illium had passed through a slit in the omentum and bent upon itself, and the intestinal structures about the incarcerated portion were somewhat softened (death having taken place in a little more than a week after the symptoms set in.)

Dr. Illoway concludes from these cases that:

1. Enemata are superior to every other method of treatment.

*a.* In the rapidity in which the cases are relieved.

*b.* And in clearly indicating whether a surgical operation will be required.

2. They are entirely safe and free from all danger, and in no way prejudice the case should a surgical operation become necessary.

It is due to Dr. Illoway to say that an operation was advised in the fatal case two days before death, and was refused. But for the length of time at which death ensued in this case, and the small amount of softening of the incarcerated portion of the intestine at this late date, we should be inclined to think that there may be an element of danger in forcible enemata in cases of some days' standing: namely, rupture of softened intestinal walls by the force of the injection and the pressure of the fluid. The fatal case must either be exceptional in regard to the degree of softening found, or we must think that the danger mentioned is comparatively slight until a longer time has elapsed. Of the two alternatives it must seem that the first should be held. It should be remembered, also, that the last injections were made on Tuesday, and then death took place on Friday; a sufficient time having elapsed for marked pathological alterations to take place. And it is entirely possible that had the forcible injections been made on Friday or the day before, the intestinal wall might have been ruptured.

However this may be, Dr. Illoway shows very conclusively that forcible injections are of great utility in promptly relieving some cases at least of one of the most unsatisfactory conditions with which the physician or surgeon has to deal. In the three favorably-terminating cases the relief was permanent and almost immediate. In one of his cases an ordinary Davidson syringe had been tried without effect before the force-pump was used; from which it would seem that sufficient force cannot be obtained from an ordinary instrument. It is scarcely necessary to say that laparotomy should be performed if the force-pump proves ineffectual with as little delay as possible. It will be remembered that in an editorial in the Journal of September 26, 1885, the treatment of intestinal obstruction was carefully discussed, and the following sentence quoted from the paper of Mr. Treeves, read before the

British Medical Association, at Cardiff: After twelve hours of treatment of acute intussusception with opium or belladonna and rest, it will be expedient to attempt reduction by means of insufflation or forcible enemata. This should be done in the first twenty-four hours, if possible, in order that laparotomy may be performed early.

**A Mode of Treating Acute Inflammation of the Knee-Joint.**—(New York Medical Journal) Mr. Richard Barwell (Lancet) advocates the treatment of acute inflammation of the knee-joint by aspirating of the joint in the following way: The knee is firmly enveloped, by preference, with a sufficiently broad band of elastic webbing; or an ordinary calico bandage will answer the purpose, care being taken to leave between two of the turns a little interval on the inner side on a level with the upper margin of the patella. At this point a tubular needle is passed into the joint. The fluid runs away, as a rule, quite easily, and often better, without the aspirator vacuum. While it flows, the hand should exercise a little pressure on the patella, effectually preventing the entrance of air, and when, the flow having ceased, the needle is withdrawn, the puncture is to be covered with sticking plaster. Pressure by means of adhesive-plaster must then be applied, and the limb placed at rest for a few days upon a splint. In traumatic cases the fluid is deeply stained with blood; in non-traumatic cases, if the evacuation is effected early, the liquid is quite clear. By this procedure the pain is immediately relieved, the temperature, if it has been high, subsides, and the patient is well in from ten days to a fortnight.

**"A Genuine Case of Hydrophobia Cured."**—Dr. H. Emmet Wootten, Kempner, Texas, (Virginia Medical Monthly) writes: On the night of April 15th, 1885, I was called to see Henry Dyer, a youth of 16, living with his parents at Butcher's Gap, Coryell county, Texas, who had been bitten by a rabid dog twenty days previous, in the calf of the right leg, taking out of the right leg, a piece of flesh one inch in length. He was having spasms, and was tied down to the floor with strong ropes. I was told that he had been snapping and foaming at the mouth for six hours, and had convulsions every thirty minutes. I at once administered twenty grains of the bromide of potassium in glycerin and water, and gave him an hypodermic injection of morphine, one half-grain every two hours, until the spasms ceased, which they did at 9:30 o'clock on the following day. I applied a plaster of sub-nitrate of bismuth one inch thick to the bitten leg and kept it there until the third day. *It brought away nearly a pint of greenish pus;* when the patient was able to leave his room. I attribute his cure to the bismuth and potash. I also gave him ten grains of calomel the second night, which operated finely. The young man is now well and has been working every day since as a farm laborer. The dog bit a cow and a sow upon the place, both of which died the tenth day. The dog was killed that day by Mr. Abner Easley, a near neighbor.

**The Therapeutic Action of Theine** (Indiana Med. Journal) has been studied by Dr. Thomas J. Mays, and in a reprint from the Medical News he concludes as follows: "From the results of the action of theine in these cases it will be seen that it is a powerful anodyne without producing any intoxication of the higher nerve centres, which is so common with morphia and other agents belonging to this class. Its influence is both quick and persistent, and it manifests an almost exclusive affinity for the sensory nerves. It relieves pain by acting from the centre towards the periphery, and showing its effects but very seldom above the seat of injection. This is precisely in harmony with what is found in experiments on lower animals. In 1-10, 1-5, and even 1-3 grain doses it is entirely free from dangerous consequences—the only inconvenience which it causes is a slight, but transient burning at the point of introduction. I use a one per cent. watery solution of Merck's preparation—ten minims of which equal 1-5 of a grain of theine. Larger doses are required in some individuals in order to bring out its characteristic action. I sincerely hope that the action of this agent will be further investigated, for if the favorable report above given is sustained, it will certainly prove itself of the greatest clinical value, and will be a vindication of the modern methods of pharmacological inquiry.

**Pichi**—(*Fabiana imbricata*) (Practitioner and News) has for years been a popular remedy among the natives of Chili in urinary disorders, jaundice, and other hepatic troubles. And is now claimed (*vide* this journal, January 23d) that it is capable of even preventing the formation of calculi, and when formed and still small, of making their discharge painless. While it is well known that no one drug can influence all caculi, pichi is deserving of use in such disorders. After what has been written of its effects in cystitis, it certainly gives sufficient promise of good to warrant a thorough trial The opportunity to do this has been given by Parke, Davis & Co., who offer samples of their preparation of the drug to physicians who wish to test it at the bedside. A drug which possesses real efficacy in either calculous, hepatic, or vesical diseases is a desideratum, and we should be glad to publish the results of the use by any of our readers of this new candidate for public favor.

**Amaurosis Due to Bromide of Potassium.**—(Recuel d' Ophthalmologie) Dr. Rubel reports, in a German Journal, a unique case of this kind. A woman, aged 23, attacked with mental disease, suffered from epilepsy, and was treated with large doses of bromide of potassium. One day she lost her sight and Dr. Rubel was consulted. An ophthalmoscope examination revealed the fact of evident paleness of the organ owing to diminution in the calibre of the retinal vessels. The bromide was discontinued and the iodide of potassium substituted. At the end of five weeks the patient recovered. Bromide of potassium was used again and blindness once more induced. The medicine discontinued, the patient again recovered.

**Symptoms Resulting from Swallowing a Caterpillar.**—(New York Medical Record) Dr. George M. Waters, of Columbus, Ohio, writes: "On November 10th I was called to prescribe for a child seven months old. From the parents I learned that the patient had been in good health previous to 2 p. m., and at that time awoke from a sound sleep with a scream and appeared to be choking. Nothing could be found in the throat to account for these peculiar actions, but considerable redness was noticed about the fauces. At 8 p. m., I found the throat intensely inflamed, a slight elevation of temperature, pulse feeble and rapid, and the patient very restless. The following morning the throat inflammation had subsided, but the temperature had reached 102°F., there were symptoms of intense gastric distress, and the face, body and upper extremities were covered with an eruption resembling a severe case of urticaria. Bismuth and oil were ordered with a mild opiate. On the morning of November 12th, the eruption, with exceptional spots, had disappeared, the temperature had fallen to 99.5°, and there were no indications of pain. The patient was much better, and well might it be, for with the first free stool was passed the body of a caterpillar an inch and a half long, and larger than the common lead pencil; the hair had been completely stripped from the body, which was complete and covered with mucous. With the two following stools came the brown hair of the caterpillar. This was followed by an inflammatory diarrhœa for three days, when the child began to improve rapidly and is now in good health."

**Pulverized Vaccine Virus.**—(Western Druggist) As long as the ideal method of vaccination, i. e., through the pure culture and subsequent inoculation of the specific germ of variola or vaccinia is unattainable, our chief aim, says the New York Medical Journal, consists in assuring ourselves of the purity of the vaccine-soil and its greatest possible freedom from other organic matters.

Dr. Hager, of Neustdt, Magdeburg, demonstrated at the last meeting of the German physicians, at Magdeburg, the process of obtaining and preparing animal lymph (Centralb. f. d. g. Ther. 1885) in the form of powder.

Dr. Reissner, of Darmsdat, was the first to introduce powdered animal lymph. According to his methods the pustules are scraped off and excised 120 hours after vaccination, then the mass is spread thinly on a glass plate, and at an ordinary temperature, brought under an exsiccator. Two days after, the dried mass is pulverized in an agate mortar; then the powder is again brought under an exsiccator and finally prepared for use by making a paste by the addition of distilled water.

**Jaundice.**—(Col. and Clin. Record) In ordinay cases of catarrhal jaundice, Dr. Neff, at the Hospital, advises the application of a blister over the region marked by a line extending from the gall bladder to the ensiform cartilage. In a few hours after the application the bile pigment disappears from the urine, and the yellow hue of trunk and body rapidly fades.

**Crotonol.**—(Popular Science News) A recent number of the Bulletin de Pharmacie contains an interesting paper, by M. G. Guerin, on "Crotonol Blisters." He calls attention to the fact, that, with the ordinary cantharides blister, we have often to contend with serious affection of the bladder. Now, the vesicating principle of croton-oil, or "crotonol," according to this writer, enables us to obtain very active blisters, which present none of the drawbacks inherent to cantharides blisters.

To obtain crotonol from croton-oil, and to use it in preparing blisters, is an easy process, and is carried out by treating the oil with alcohol of ninety degress. Equal parts of croton-oil and alcohol are shaken up together in a well-stoppered flask, and then allowed to remain perfectly quiet for several hours. Two distinct layers are formed. The alcoholic layer contains the whole, or nearly so, of the active principle. This layer is carefully drawn off from the other, and placed in a porcelain dish over a water-bath until the alcohol has evaporated.

In this manner an oily liquid is obtained, which is rather more viscous than the original croton-oil, and very much more active. This is the product that has been named crotonol. In order to apply it to the vesicating surfaces, pieces of linen are cut to the proper dimensions, and are fixed to the diachylon by simple pressure of the hands. Sufficient crotonol is then poured on to moisten the linen.

**Choera.**—(Philadelphia Medical Times) M. Joffroy treats chorea successfully with chloral hydrate and the wet sheet. The dose of chloral, for patients over ten years of age, would be one drachm, in three doses, given after meals; fifteen grains about seven in the morning, fifteen at noon, and thirty at about six o'clock in the evening. In children of six or seven years the dose must be reduced; but enough should be given to induce sleep fifteen minutes after the administration of each dose. If the choric movements are very decided, it is recommended to wrap the child in a wet sheet for half an hour night and morning, and afterwards to have the body well rubbed with the palm of the hand. Eighteen cases reported by M. Sarie show that this treatment is of decided value. It is claimed that it reduces not only the duration, but also the intensity of the disease. In the majority of cases it terminates in four or six weeks.

**Cocaine in Inflamed Nipples.**—(Chicago Medical Times) Urnna says (Wiener Medical Wochenschrieft) that muriate of cocaine is without a rival in the treatment of sore nipples. The obstinacy of fissured nipples, when the child continues to nurse is almost proverbial. Cocaine affords prompt relief. All cases treated by the author terminated successfully. The nipple is brushed with a one-half per cent. solution every ten minutes while the child is at the breast. The pain disappears immediately and within two days the nipple is well. There is no danger of its absorption by the child—in fact it may be of benefit where the child is irritable and restless

**Action of Pilocarpine and Atropine.**—(Medical World) M. Judic, in a communication on the influence of pilocarpine and atropine on perspiration, made before the Biological Society of Paris, stated that if a dog's spinal cord be cut between the eighth and ninth dorsal vetebræ, its paws become the seat of intense perspiration. This appears to prove that there is a spinal nerve-center, which regulates the secretion of sweat. After dividing the sciatic nerve, if the peripheral end be stimulated, the corresponding paw perspires profusely. The sciatic nerve is simply a transmitting agent; it establishes communication between the medullary and the peripheral nerve-centers. If, instead of stimulating the peripheral end of the sciatic, the nerve be left intact, the pilocarpine be administered to the animal, the perspiration is equally intense. If the nerve be cut and pilocarpine administered, the perspiration is normal. It may, therefore, be concluded, that pilocarpine does not act on the glandular elements, but on the nervous system. Atropine produces the opposite effect to that provoked by pilocarpine.

**Premature Labor by Electricity.**—M. Bayer, of Strasburg, has had, during the past year, four opportunities to test the efficacy of the constant electric current in inducing premature labor. In all these cases the electricity developed uterine contractions, dilated the os, and overcame the constriction of the neck, but the action was found to be unequal. The best effects were had in subjects whose uteri were powerfully muscular, the cervices not being too rigid.

**Reduction of Dislocations by Pressure.**—(Charles Young, in British Medical Journal) I have twice reduced dislocations of the thumb by grasping the hand with my two hands, and pressing with my thumbs on the dislocated articular surfaces; and two or three times I have reduced partial dislocations of the shoulder forward by raising the arm with one hand and pressing back the head of the bone with the other, standing behind the patient.

**Hamamelis in Prostatic Enlargement.**—(New York Medical Journal) Dr. Mackenzie says, in the British Medical Journal, that irrigation of the bladder with a mixture of a drachm of the tincture of hamamelis, half a drachm of carbolic acid, and about twenty-five ounces of warm water, arrested periodical hemorrhages from the urinary passages and the rectum in a case of prostatic enlargement under his care, and so reduced the congestion as to enable him to discontinue the use of the catheter.

**Painless Tooth Extraction.**—(N. E. Medical Monthly) Dr. Hepburn, in the Independent Practitioner says that teeth can be extracted without pain in the following manner: The tincture of purified extract of cannabis indica is diluted with from three to five parts of water. This is applied to the gums by rubbing with the finger dampened with the solution. The forceps are also dipped into the solution before applying them to the teeth.

